# Tribally-led mobile outreach: improving access to harm reduction services in one rural reservation community

**DOI:** 10.3389/fpubh.2024.1383729

**Published:** 2024-05-16

**Authors:** Allyson Kelley, Kellie Webb, Katherine Hirchak, Morgan Witzel, Kelsey Bajet, Sadie Posey

**Affiliations:** ^1^Allyson Kelley and Associates PLLC, Sisters, OR, United States; ^2^Doya Natsu Healing Center, Eastern Shoshone Tribe, Fort Washakie, WY, United States; ^3^PRISM Collaborative, Department of Community and Behavioral Health, Elson S. Floyd College of Medicine, Washington State University, Spokane, WA, United States

**Keywords:** harm reduction, mobile outreach van, American Indian, reservation, re-aim, evaluation

## Abstract

American Indian and Alaska Native populations in the United States face significant disparities related to opioid use disorder and opioid-related mortality. Inequitable access to medications and harm reduction strategies due to structural, societal, and geographical factors prevent Tribal communities from obtaining needed services, and further contribute to the opioid epidemic. One Tribal Healing Center in the Rocky Mountain region identified mobile outreach to build upon existing opioid prevention, treatment, and harm reduction efforts. The Healing Center purchased a mobile outreach vehicle and worked with a combination of clinical staff, peer recovery support specialists, and Tribal elders to reach identified high-risk areas on the reservation. As of December 2023, the mobile outreach vehicle has disseminated 150 Narcan kits, 150 Fentanyl testing strips, 20 self-care kits, and 500 brochures detailing Healing Center services. Preliminary results from this formative evaluation demonstrate the success of MOV efforts and the process required to purchase and launch an MOV campaign.

## 1 Introduction

American Indians and Alaska Native (AI/AN) people experience disparities in drug and opioid-involved overdose mortality rates when compared with other racial and ethnic groups (1–3). Between 1999 and 2019, opioid-involved overdose mortality rates among AI/AN communities have seen a significant increase from 5.2 to 33.9 per 100,000 AI/AN persons (4). One national study calls attention to the devastating and disproportionate impacts of opioid overdose deaths on Tribal lands; from 2006 to 2014, AI/AN adults were 50% more likely to die of an opioid overdose than non-AI/ANs (5). Historical and political factors such as boarding schools, forced relocation, unresolved trauma, and discrimination contribute to health inequities and increased mortality among AI/AN people (6). Structural racism, including poor working conditions, lack of economic opportunities, and limited social capital in communities, are the primary drivers of opioid misuse (7). These factors, coupled with unregulated opioid prescribing practices, systemic issues with healthcare facilities and health service delivery in Indian country, and unfair marketing practices targeting AI/AN populations, have resulted in a significant drug crisis (8).

Western models of opioid prevention and treatment are often applied to AI/AN populations in an effort to address the current epidemic (9). Medications for Opioid Use Disorder (MOUD), such as buprenorphine/naloxone, is one intervention approach used in the US general population and in AI/AN communities (10). However, AI/AN populations do not have equitable access to medications and harm-reduction strategies (11). Structural discrimination, oppression, marginalization, rurality, and differences in beliefs about access to Naloxone and harm reduction make it difficult to address the opioid crisis using harm reduction in AI/AN communities. Healing advocates are calling for harm reduction approaches that integrate cultural beliefs, practices, community, and family to create conditions that support holistic wellness and connections (12).

The Substance Abuse and Mental Health Services Administration (SAMHSA) has expanded several opioid-related grants for communities and health centers that fund the implementation of evidence-based treatment and prevention programs (13). SAMHSA grants have increased access to MOUD and increased funding, totaling over $6.6 billion in 2022.

One approach to increasing harm reduction strategies that has been successful or is gaining support within diverse communities is mobile outreach. Mobile outreach programs take information, education, services, and support to individuals who are at risk for drug overdose. Mobile outreach services occur in non-traditional locations and are designed to meet people where they are, with the services that they need. Peer support specialists, individuals with the lived experiences of recovery, and community health workers often lead mobile outreach efforts because of their expert knowledge of community conditions and needs. Efforts to address OUD using mobile outreach and harm reduction approaches in AI/AN populations are somewhat limited. The National Institutes of Health (NIH) HEAL Initiative, launched in 2019, aims to integrate medication-based treatment into primary health care and addiction treatment settings using culturally appropriate methods (14). No outcomes from this research have been published. The Indian Health Service (IHS), the primary healthcare provider for federally recognized Tribal members and their descendants, has developed several resources, funding opportunities, training, and recommendations for addressing OUD and pain in AI/AN populations (15). IHS work has resulted in increased funding to AI/AN providers and community health programs, but individual and community-level outcomes have not been published.

There is limited to no information available about how to conduct mobile outreach in rural Tribal reservation communities to address the opioid epidemic while promoting community wellness. Mobile outreach, often called mobile units or MOVs, is used by health professionals to provide a variety of health-related services to hard-to-reach populations (16). Increasingly, mobile outreach is being used to improve access to MOUD, and harm reduction services like syringe exchange, naloxone distribution, and referrals to MOUD programs (17). This paper fills an important gap in the literature by documenting the process of purchasing a MOV, planning outreach, implementing MOV outreach, creating an evaluation protocol, and identifying some early lessons learned from the process.

## 2 Context

Surveillance of suspected overdoses, fatal overdoses, and Naloxone administrations are recorded by first responders and law enforcement officers across jurisdictions, including counties where reservations are located. The Overdose Detection Mapping Application (ODMAP) is helpful in targeting MOV outreach efforts.

The Tribe is located in the Rocky Mountain region. With more than 2.2 million acres of rural land, the reservation is home to 12,500 Tribal members. The Healing Center is a State-certified provider of substance use services as an outpatient treatment provider. The Healing Center provides integrated treatment services for substance use, mental health, and cultural resilience. Its mission is to provide community-based, integrated prevention and treatment services that encompass core cultural values and the Medicine Wheel teachings for individuals, families, and the community. The Healing Center leads several substance abuse and opioid-related treatment grants from SAMSHA. In-person outpatient and healing services are offered in two locations, one in a non-reservation border town and the other in a private room in the reservation-based Healing Center. Services are open to anyone in the community, including non-AI/AN populations and individuals without OUD or chronic pain issues. This approach is consistent with Tribal values of inclusivity, kinship systems, and community. Utilizing a multi-pronged outreach approach, the Healing Center promotes recruitment and the many pathways to healing on social media, printed posters, email communications, and word-of-mouth.

### 2.1 Creation of a harm reduction approach using a mobile outreach van (MOV)

In 2022, the Healing Center received grant funding from SAMHSA to provide MOUD services in the community. This grant provided the necessary funding to purchase an MOV. The MOV was designed to augment services and promote the use of MOUD in hard-to-reach populations on the reservation. MOV services are designed and offered using a harm-reduction model based on the assumption that return to the use of substances is part of recovery and that everyone has a place in the sacred circle of life. MOV efforts build on existing harm reduction approaches implemented by the Healing Center, which include Naloxone poisoning kits/training, Fentanyl test strip distribution, Medicine Wheel teachings (e.g., a whole person approach to health that includes the physical, mental, emotional, and spiritual), holistic self-care, sweat lodge, talking circle, storytelling, complementary alternative medicine, and contingency management.

Designing and purchasing the MOV took 11 months and started in July 2022. The Healing Center utilized grant funds to purchase the vehicle. This required extensive planning and the following four steps.

**Step 1.** Awareness of federal purchasing requirements and bids.

The Healing Center was required to get bids from at least 3 sellers showing the cost of an MOV. Bids received ranged from $130,000 to $250,000. Other requirements were to list the number of passengers that could fit in the unit, specify how long it would take to procure and receive the van, and estimate the number of people that would be served by the MOV. The final question was to explain the effect on the program if the Healing Center is unable to procure the vehicle.

**Step 2.** Procurement and preparing for delivery.

The Healing Center selected the bid that was the lowest because there were limited grant funds available to purchase the MOV. Once the bid was accepted, the MOV company sent an invoice with the specs of the MOV. The invoice was approved by the Tribal Business Council, and funds were allocated from the SAMHSA grant for purchase. These were required before production could take place. The MOV is wrapped with the Healing Center’s logo and contact information. This was the final step in the production process after the MOV was built. The Healing Center worked with a team of staff and behavioral health specialists to plan outreach methods upon delivery. The Healing Center also created a plan for where the MOV would be located, who would maintain the MOV, insurance requirements, gas, stocking the MOV with supplies, and other details.

**Step 3.** Planning and implementing harm reduction outreach.

Existing data from ODMAP is used by the Healing Center to inform community-based harm reduction MOV efforts. The Healing Center works with the staff, behavioral health team, and community members to identify events, locations, and areas where MOV could benefit the community. Discussions also centered around the kinds of supplies and harm-reduction materials to share during outreach events, for example, self-care kits in the winter months with warm clothing options and basic hygiene items. The MOV is parked at the Healing Center when it is not in use. Regular maintenance, cleaning, and stocking are completed regularly by the Healing Center staff and local businesses.

**Step 4.** Evaluating MOV harm reduction outreach.

The Healing Center works with an external team for evaluation purposes. They utilized principles of community-based participatory research (CBPR) informed by Indigenous and community knowledge to design and implement the MOV evaluation (18). The evaluation team works in collaboration with the Healing Center and staff where co-learning, empowerment, and reflection are evident. Formative evaluation of the MOV is in its early stages, and the team selected the Re-AIM framework informed by Indigenous values to evaluate MOV harm reduction efforts.

### 2.2 Naming and branding the MOV initiative

**Figure 1:**
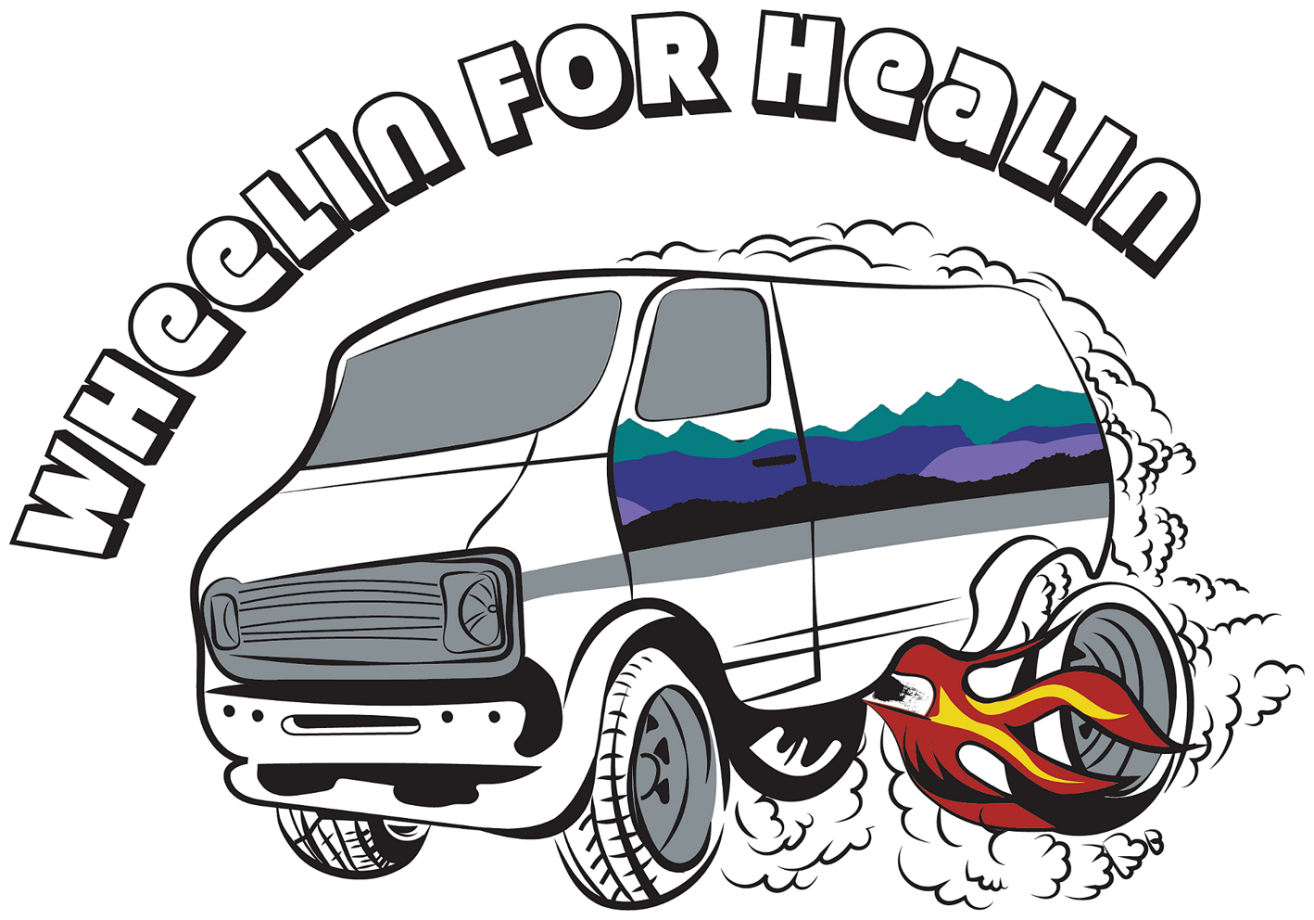
Wheelin for Healin MOV logo.

The Healing Center partnered with the local Indian Health Service Behavioral Health team to design and name the MOV initiative “Wheelin for Healin” (see Figure 1).

Working in partnership with local officials and the ODMAP data surveillance system, Wheelin for Healin aims to identify high-risk areas on the reservation and bring harm reduction services to these areas. Wheelin for Healin utilizes a combination of clinical staff, peer recovery support specialists, and Tribal elders and knowledge keepers to reach these areas. Additional services are provided by Tribal medical staff, pharmacists, and public health nurses (Table 1).

**Table 1:** Services provided by the reservation-based Healing Center.

Holistic Care based on the Medicine Wheel-spiritual, mental, physical, emotional
Addiction care
Harm reduction services
Complementary alternative medicine
Talking circles
Elder storytelling
Traditional plant and medicine preparations

The Healing Center worked with an external evaluation team to create culturally-centered, community-driven evaluation approaches that show value and tell the story of prevention and healing on the reservation. All evaluation procedures were approved by the Tribal Business Council, which serves as the regulatory authority for all data collection and evaluation/research on the reservation. Quantitative data came from Accucare, the Healing Center’s electronic health record. Qualitative data came from meetings with the evaluation team and the Healing Center staff during the planning and implementation phases. Quantitative data were analyzed using Excel and basic frequency counts. Qualitative data were analyzed using content analysis methods informed by recovery and Indigenous approaches (19).

The next section provides preliminary findings from the MOV evaluation in the first 6 months of the project and a framework for future data collection using the RE-AIM approach (20).

## 3 Results

Combined, the evaluation team explored notes, meeting minutes, emails, and electronic health record reports from the Healing Center from July 2023 to December 2023. Results from the quantitative demonstrate the early success of the MOV outreach to address poisoning and promote harm reduction strategies. Results from the qualitative data demonstrate early lessons learned that will be applied to future outreach and evaluation. For example, in one instance, the Healing Center staff parked the MOV at a GONA, but nobody visited the MOV. Another outreach in the village had limited people; the staff felt it was too early in the morning and recommended going back at a later time when people were awake. Other recommendations include providing snacks and promoting MOV events on social media (website, Facebook).

The MOV unit is also used at various community outreach events, conferences, powwows, and recovery-related activities. The Healing Center logo and MOV message of Wheelin for Healin support awareness of harm reduction efforts and recovery services available in the community. As of December 2023, the MOV has disseminated 150 Narcan kits, 150 Fentanyl testing strips, 500 brochures about Healing Center services available, and 50 self-care kits. Billboards are located on the reservation in bordering towns; these promote the Healing Center MOV branding and reach more than 10,000 people in the area (Table 2).

**Table 2:** Characteristics of services provided on reservation via the MOV from June 2023 to December 2023.

Event name	Location (rural or isolated rural)	Staffing	Reach	Services/resources provided
477/TANF event	Rural	Healing Center	100 people	Promote the brand, increase awareness of MOV
Gathering of native Americans (GONA)	Rural	Healing Center	10	Promote brand, increase awareness of MOV
Village #1 outreach	Isolated rural	Psychiatric nurse practitioner, pharmacist, Healing Center	7 cars drove by no stops 10 people received supplies	Narcan and testing strips Self-care kits Program information
Village #2 outreach	Isolated rural	Healing Center	3 cars drove by no stops 4 people received supplies	Narcan and testing strips Self-care kits Program information
Various tribal events	Rural	Healing Center	500 people received supplies	Program information

#represents the number or frequency.

### 3.1 RE-AIM

An evaluation protocol was developed for the MOV using the RE-AIM framework. RE-AIM is a framework that has utility in the evaluation and research of implementation strategies in substance use treatment contexts with and by Indigenous populations (21). Table 3 outlines how MOV efforts will be used to explore RE-AIM categories in the future.

**Table 3:** RE-AIM evaluation protocol of MOV.

Re-AIM category	Measure	Data collection	Frequency
Reach	# of people reached	Sign-in sheets	As needed, all MOV outreach
Reach	# of harm reduction resources distributed	Count of resources available and remaining	When disseminating resources
Effectiveness	# of poisonings on the reservation by month	ODMAP data	Monthly
Adoption	# staff involved in MOV	Timesheets	Monthly
Adoption	% increase in community members accessing resources	Count of people in communities using resources	As needed
Implementation	# referrals to healing center/behavioral health	Electronic health record intake forms	As needed
Maintenance	% reduction in poisoning on reservation annually	ODMAP data	Annually

#represents the number or frequency.

Future evaluation efforts will focus on documenting the kinds of services needed in villages and how to create referral processes in these communities to address gaps in the current service delivery system, for example, transportation. Evaluation in the future will also explore how often Naloxone kits are used, the kinds of harm reduction services that people need the most, and bringing CAM services to villages via the MOV.

## 4 Discussion

This case study discussed one rural reservation community’s efforts to develop harm reduction strategies to address the opioid epidemic in a culturally centered manner. Protocols, plans, and outreach began in the summer of 2023 and are ongoing. Our study also described the first MOV in the region to promote a harm reduction model and services. Tribal communities like this one positively impact their own health and exert Tribal sovereignty through MOV initiatives and culturally centered harm reduction strategies. A strength of this MOV approach is that it utilizes the RE-AIM framework and builds on previous evaluations and research conducted in the community to guide the approach (18, 20). Data are collected locally during MOV outreach and information is consistently used for improving MOV strategies. Data collection often involves a pen and paper survey, administrative notes, and an online survey created in Qualtrics. In this study, the highest number of people were reached during Tribal events. This is because there is an audience of people in one location in contrast to a small village outreach in a community where just a few people come out of their homes to learn about MOV efforts. Cultural events are another key component to the increased engagement. Future MOV outreach will be explored at powwows, health fairs and other opportunities where vendors are welcome. Time of day was also determined to impact engagement. Activities completed in the afternoon had better reach than morning activities. In addition, creative community marketing and engagement strategies will be employed using the Healing Center podcast, website, and social media. The monthly program newsletter will also provide updates on MOV activities. Materials will continue to be shared with partnering agencies.

MOV outreach addresses a community identified need of transportation and access to services. Similar to other MOV initiatives, plans to increase interest in MOV outreach will focus on low-barrier efforts. Intentional selection of staffing alongside a variety of MOV services has increased engagement in other rural communities (22). Additional services will be integrated in the future consistent with the literature, these might include employment and insurance services, recovery and peer-support, distribution of food and other necessities, and addressing co-substance use through the delivery of other culturally centered evidence-based treatments (e.g., contingency management for stimulant use). In the Pacific Northwest region, Tribal programs have increased health and well-being through their harm reduction strategies. For example, efforts by the Swinomish Indian Tribal Community have included the development of a program to address the opioid epidemic that centered on community values, resulting in a 50% reduction in opioid-related mortality (23). Other effective community-based solutions include the Southcentral Foundation in Alaska. A relationship-based approach was implemented to treat opioid use disorder for the 229 Tribal communities served within the large healthcare system. Promising outcomes included a 45% reduction in opioid prescriptions within a two-year period (24).

As planning and outreach continue, the Healing Center will look to other regions with MOVs to learn and grow. The Healing Center has continued MOV outreach and evaluation using the RE-AIM framework and protocol outlined in this paper. This will be in addition to expanding access to a variety of services for individuals in need. Partnerships are essential to the success of harm reduction efforts. The work with the Indian Health Service, CAM providers, Tribal Elders, and knowledge keepers will inform future work. Preliminary results from this formative evaluation demonstrate the success of MOV efforts and the process required to purchase and launch an MOV campaign. Continued surveillance efforts from ODMAP and data sharing will support focused outreach in high-risk areas on the reservation. Future work may consider how to provide primary care services, screenings, referrals, long-term impact on opioid related mortality and infections, and follow-up to the Healing Center from MOV outreach.

To maximize MOV outreach in rural areas, continued funding of harm reduction approaches that address barriers like transportation, access, and discrimination are necessary. Harm reduction supplies like free Naloxone, Fentanyl test strips, safer sex supplies, educational resources, and referrals encourage rural citizens to practice safe behaviors. Rural communities and reservations throughout America have varying views about harm reduction strategies; MOV outreach promotes a non-judgmental and non-punitive approach, people who know the community and have the lived experience of recovery are the most effective and trusted individuals to lead such efforts in rural locations.

## 5 Conclusion

Harm reduction strategies encompass a variety of methods aimed at improving public health and limiting negative consequences associated with substance use. A holistic harm reduction approach will continue to be led by the Healing Center, located in the community and supported by the Tribal Business Council. Healing Center staff and partners will work to create innovative, community-driven public health equity-focused strategies that prevent the use of substances while reducing poisoning (overdose) risks. The Healing Center’s MOV program brings much-needed services into the community and out of the clinical setting. The decolonized approach to outreach promotes a message of hope, longevity, and community. This paper provides a starting point for information about how to conduct mobile outreach in rural Tribal reservation communities to address the opioid epidemic while promoting community wellness.

## 6 Acknowledgement of conceptual or methodological constraints

While the MOV approach and preliminary evaluation data show promising results, more MOV is necessary to fully understand the best harm reduction strategies in this reservation community. Follow-up strategies are needed to document the number of people who seek services as a result of MOV outreach. Additional considerations of practical costs like miles traveled per day, gas mileage, staff time, and maintenance are necessary to sustain MOV efforts into the future. MOV data presented here is based on a small sample and window of time (6 months); as MOV outreach continues, the Healing Center will collect additional data that documents all RE-AIM components and effectiveness.

## Data availability statement

The datasets presented in this article are not readily available because the datasets for this article are not publicly available due to concerns regarding participant/patient anonymity. Requests to access the datasets should be directed to the corresponding author. Requests to access the datasets should be directed to ak@allysonkelleypllc.com.

## Author contributions

AK: Writing – review & editing, Writing – original draft, Project administration, Methodology, Formal analysis, Data curation, Conceptualization. KW: Writing – review & editing, Writing – original draft, Project administration, Methodology, Conceptualization. KH: Writing – review & editing, Writing – original draft. MW: Writing – review & editing. KB: Writing – review & editing. SP: Writing – review & editing.
